# Suppression of Basophil Histamine Release and Other IgE-dependent Responses in Childhood *Schistosoma mansoni*/hookworm Coinfection

**DOI:** 10.1093/infdis/jiu234

**Published:** 2014-04-29

**Authors:** Angela Pinot de Moira, Colin M. Fitzsimmons, Frances M. Jones, Shona Wilson, Pierre Cahen, Edridah Tukahebwa, Harriet Mpairwe, Joseph K. Mwatha, Jeffrey M. Bethony, Per S. Skov, Narcis B. Kabatereine, David W. Dunne

**Affiliations:** 1 Department of Pathology, University of Cambridge, United Kingdom; 2 Vector Control Division, Ministry of Health, Kampala, Uganda; 3 MRC/UVRI Uganda Research Unit on AIDS, Entebbe; 4 Centre for Biotechnology Research and Development, Kenya Medical Research Institute, Nairobi; 5 Department of Microbiology, Immunology, and Tropical Medicine, George Washington University, DC; 6 RefLab ApS, Copenhagen, Denmark

**Keywords:** *Schistosoma mansoni*, schistosomiasis, hookworm, helminth infection, allergy, skin prick test, wheeze, basophils, histamine release, house dust mite

## Abstract

***Background.*** The poor correlation between allergen-specific immunoglobulin E (asIgE) and clinical signs of allergy in helminth infected populations suggests that helminth infections could protect against allergy by uncoupling asIgE from its effector mechanisms. We investigated this hypothesis in Ugandan schoolchildren coinfected with *Schistosoma mansoni* and hookworm.

***Methods.*** Skin prick test (SPT) sensitivity to house dust mite allergen (HDM) and current wheeze were assessed pre-anthelmintic treatment. Nonspecific (anti-IgE), helminth-specific, and HDM-allergen-specific basophil histamine release (HR), plus helminth- and HDM-specific IgE and IgG4 responses were measured pre- and post-treatment.

***Results.*** Nonspecific- and helminth-specific-HR, and associations between helminth-specific IgE and helminth-specific HR increased post-treatment. Hookworm infection appeared to modify the relationship between circulating levels of HDM-IgE and HR: a significant positive association was observed among children without detectable hookworm infection, but no association was observed among infected children. In addition, hookworm infection was associated with a significantly reduced risk of wheeze, and IgG4 to somatic adult hookworm antigen with a reduced risk of HDM-SPT sensitivity. There was no evidence for *S. mansoni* infection having a similar suppressive effect on HDM-HR or symptoms of allergy.

***Conclusions.*** Basophil responsiveness appears suppressed during chronic helminth infection; at least in hookworm infection, this suppression may protect against allergy.

Prevalence of allergy has increased markedly since the beginning of the 20th century [1]. Epidemiological observations suggest that this may be related to environmental and lifestyle changes associated with urbanization, as allergies appear more common in developed than in developing countries [2], and even within countries there appears to be distinct division between rural and urban environments [3–5]. Furthermore, as urbanization increases in developing countries, the burden of allergy appears also to be increasing [6].

Allergic (immunoglobulin E [IgE]-mediated) disorders are usually confirmed by measuring skin prick test (SPT) sensitivity or allergen-specific IgE (asIgE) [7]. However, these 2 measures are often poorly correlated and weakly specific for clinical phenotypes. In a large-scale study conducted by the International Study of Asthma and Allergy in Childhood (ISAAC) group, this discordance varied dramatically by geography, with a strong negative correlation with gross national income per capita (GNI) [8]. Furthermore, although no overall association between asIgE and GNI was observed, there was a weak positive association between SPT and GNI. Similar observations have been made elsewhere [9]. One explanation could be the greater prevalence of helminth infection in lower GNI countries. Infection with helminths has similarly been associated with discordance between asIgE and SPT sensitivity [10–12], and there is growing evidence that at least some species of helminth protect against allergy [13, 14].

Immune responses seen in allergy and during helminth infection are markedly similar. Both are Th2-biased, with elevated levels of interleukin 4, interleukin 5, and interleukin 13 cytokines, IgE and IgE-effector cells, such as basophils and eosinophils. In helminthiasis, specific IgE responses are associated with immunity to infection [15–18]; however, this immunity is only partial and appears finely balanced between the IgE-effector mechanisms of IgE and IgG4 down-regulatory effects [16, 18–21], which is able to block IgE antigen binding and suppress IgE-effector cells by engaging FcγRIIB [22]. Regulatory cells and cytokines such as Tregs, interleukin 10, and transforming growth factor β are also up-regulated in helminthiasis [23], together preventing allergic inflammation.

We hypothesize that immunoregulatory responses seen in helminth infection reduce the response of IgE-effector cells such as basophils to IgE-mediated activation, uncoupling IgE from its effector mechanisms, resulting in suppressed responses to both parasite and nonparasite antigens. Here, we test this hypothesis in schoolchildren from an area co-endemic for *Schistosoma mansoni* and hookworm by (1) measuring changes in specific and nonspecific histamine release (HR) in whole blood 8 weeks after concurrent praziquantel and albendazole administration to treat schistosomiasis and hookworm, respectively, and (2) examining associations between antigen-specific-IgE and antigen-specific-HR, and how these are influenced by helminth infection. We further hypothesize that the uncoupling of asIgE from its effector mechanisms has downstream effects to limit allergy; we test this by measuring house dust mite skin-prick-test (HDM-SPT) sensitivity and current wheeze, and how these are influenced by current helminth infection.

## MATERIALS AND METHODS

### Study Population

This study was conducted in Bwondha Village, on the shoreline of Lake Victoria, Mayuge District, Uganda. Ethical clearance was obtained from the Uganda National Council of Science and Technology. Full study details are given elsewhere [24]. Briefly, a register of all children (n = 795) attending Bwondha Primary School was drawn up and from this, 350 children aged 7–16 years were selected by simple random sampling. Written informed consent was obtained from all parents/guardians of sampled individuals who agreed to participate. Questionnaires were administered to the most relevant parent/guardian, recording household socioeconomic characteristics (eg, parental occupation and education, house construction, water sources, sanitation, asset ownership). Wheeze in the last 12 months was collected by questions adapted from the ISAAC questionnaire [2]; recall bias is likely to minimal within 12 months. All questionnaires were translated and administered in Luganda, the predominant language in Bwondha.

### Treatment, Blood and Stool Collection

In July 2010, 350 children were treated once with albendazole (400 mg) and twice with praziquantel (40 mg/kg bodyweight, 1 week apart). Venous blood samples (5 mL) were collected from children pretreatment and 8 weeks post-treatment. Three stool samples were collected on 3 consecutive days, pretreatment and 5 weeks post-treatment, to detect treatment failure/noncompliance and determine treatment efficacy. Two 50 mg Kato-Katz thick-smear slides were prepared from each sample and examined microscopically (within 30 minutes of preparation for hookworm quantification). Of the 350 children recruited, 240 completed the study. Loss to follow-up was mainly related to the transient nature of the study population.

### Parasite and Environmental Antigens

Crude somatic adult hookworm antigen extract (AHW) was prepared from *Ancylostoma caninum*, from canines. A Puerto Rican strain of *S. mansoni*, maintained in outbred mice and *Biomphalaria glabrata*, was used for production of schistosome crude antigens. Adult worms were recovered from mice 6 weeks after infection, and parasite eggs isolated from liver tissue. Soluble worm antigen (SWA) was prepared from frozen worms, and saline-soluble egg antigen (SEA) from frozen eggs, as described elsewhere [15, 25]. House dust mite antigen (HDM) was an equal mixture of *Dermatophagoides farinae* and *D. pteronyssinus*; Greer, Lenoir, USA).

### Skin Prick Testing

Skin prick testing for HDM sensitivity was conducted pretreatment using standard procedures [26]. Histamine was used as a positive control and saline solution as a negative control. A mean wheal diameter ≥3 mm compared to the negative control was defined as a positive reaction, with the reading taken 15 minutes after pricking allergen onto the volar side of the forearm. HDM allergen, controls, and lancets were obtained from ALK-Abello (Horsholm, Denmark).

### Histamine Release Assays

Histamine-binding glass fiber-coated microtitre plates (RefLab ApS, Copenhagen, Denmark) were coated with 25 µL/well of antigen in triplicate, at concentrations of 7 µg/mL (α-IgE, Dako, Glostrop, Denmark), 20 µg/mL (SEA), 20 µg/mL (SWA), and 7 µg/mL (HDM), and with histamine standards, in triplicate, at 80, 50, 40, 30, and 10 µg/mL, diluted in water containing 5% glycerol. Coated plates were dried for 6 hours at 37°C, then packed and sealed for use in Bwondha. 25 µL/well of AHW antigen at 20 µg/mL diluted in PIPES Buffer (RefLab ApS) were added in triplicate to plates in the field.

HR assays followed a similar protocol to that described previously [27] but with the washing stage omitted, thus measuring direct HR by unwashed whole blood. Briefly, 25 µL of PIPES buffer was added to each well of glass fiber-coated microtitre antigen-coated plates to dissolve antigen, followed by 25 µL of unwashed whole blood. Plates were incubated for 1 hour at 37°C to allow HR and then washed with distilled H_2_O. Plates were dried, away from direct sunlight for 24 hours, and then stored in the dark at room temperature before being shipped to Cambridge, United Kingdom, for histamine analysis. Histamine was measured by spectrofluorometry as described elsewhere [28], and results were expressed as ng HR/mL blood.

### Antibody Assays

Plasma was separated from venous blood samples and stored at −80°C until required. IgE and IgG4 levels to AHW, SEA, SWA, and HDM were measured by enzyme-linked immunosorbent assay (ELISA) as described elsewhere [29]. Briefly, antigen, 15 µL/well, was placed in 384-well plates at saturation coating concentrations of 5 µg/mL (AHW), 1.2 µg/mL (SEA), 8 µg/mL (SWA), and 2 µg/mL (HDM) as determined by titration. 15 µL of sample plasma and noninfected plasma controls were assayed in duplicate at dilutions of 1/20 (IgE) and 1/200 (IgG4). A 3-fold serial dilution of purified human IgG4 (Sigma-Aldrich) or IgE myeloma (Calbiochem) was added directly to each plate to form a 14-point standard curve, starting at 30 µg/mL. For schistosome and HDM assays, detection was as described in Fitzsimmons et al [29]. For AHW assays, detection was as described in Pinot de Moira et al [24].

For total-IgE (tIgE) ELISAs, microplates were coated in carbonate/bicarbonate coating buffer pH9.6 with 15 µL/well of mouse monoclonal anti-human IgE antibody, clone G7-18 at 2 µg/mL (BD Pharmingen, Oxford, UK) and monoclonal mouse anti-human biotinylated IgE (clone G7-26) (BD Pharmingen) was used for detection. Otherwise assays were as described above.

### Statistical Analysis

Infection intensity was expressed as mean egg count per gram (epg). Due to overdispersion, epg values were transformed to the logarithm, ln(epg + 1) for statistical analysis. Details relating to household economic characteristics were used to construct a proxy measure of socioeconomic status (SES), using principle component analysis, as described elsewhere [30, 31].

Detection thresholds for positive helminth-specific isotype responses were calculated as the mean plus 3 standard deviations (SD) of noninfected European control plasma samples, and for tIgE and anti-HDM responses, as the mean plus 3 SDs of blank wells. Histamine readings were analyzed after subtracting spontaneous HR. Due to overdispersion, antibody and histamine readings were log-transformed with an integer giving the best transformation, *k*, added/subtracted, and geometric means (GM) calculated.

Significant changes between HR pre- and post-treatment were determined using paired *t*-tests. Associations between antigen-specific HR and antigen-specific IgE were explored using linear regression analysis; the significance of any change in these associations post-treatment was tested using 2-level linear regression models to allow for correlation within children, with time as “level 1” units and child as “level 2” units, with an interaction term fitted between time and antigen-specific IgE.

Two further analyses were conducted on HDM-HR data: (i) the effect modifying behavior of hookworm infection or *S. mansoni* infection intensity on associations between HDM-IgE and HDM-HR were analyzed using pretreatment data in linear regression analysis, with an interaction term between HDM-IgE and hookworm prevalence or ln(*S. mansoni* epg + 1); (ii) change between HDM-HR pre- and post-treatment was calculated as the difference between logarithms of post- and pretreatment HR; linear regression models were then constructed to analyze how post-treatment HDM-IgE and pretreatment infection influenced this change, with an interaction term between HDM-IgE and hookworm prevalence or ln(*S. mansoni* epg + 1). For both of these analyses hookworm was included as a binary variable for ease of interpretation and to ensure correct specification of the model; the high prevalence of schistosomiasis gave insufficient power to model *S. mansoni* as a binary variable, therefore, it was included as a continuous variable.

Pretreatment helminth infection and antibody response effects on HDM-SPT sensitivity and current wheeze was analyzed using logistic regression. All regression models were adjusted for age, sex, and SES; for this, age was classified into 2 binary groups of roughly equal size (7–10 years, 11–16 years) and SES fitted as the logarithm of the continuous variable.

Multilevel models were ﬁtted in MLwiN (Bristol University, UK); all other analyses were conducted using Stata 12.1 (StataCorp, USA).

## RESULTS

### Study Characteristics

As detailed elsewhere [24], of the 350 children selected for recruitment, 240 were enrolled in the study and provided assent, donated at least 2 pretreatment stool samples, blood samples pre- and 8-weeks post-treatment, and complied with treatment procedures. The comparative characteristics of these 240 children and original 350 children selected are given in Table 1. *S. mansoni* and hookworm infection prevalence was 93.8% (95% confidence interval [CI], 89.9%, 96.5%) and 80.4% (95% CI, 74.8%, 85.2%), respectively; overall GM infection intensity was 172.73 (95% CI, 131.61, 226.72) for *S. mansoni* and 71.75 (95% CI, 51.52, 99.90) for hookworm.

**Table 1.** Pretreatment Characteristics of Participating Children

	N (%)	
	Original Sample (n = 350)	Participants^a^ (n = 240)
Age, years		
7–8	104 (29.7)	61 (25.4)
9–10	80 (22.9)	57 (23.8)
11–12	106 (30.3)	82 (34.2)
13–16	60 (17.1)	40 (16.7)
Sex		
Male	169 (48.3)	117 (48.8)
Female	181 (51.7)	123 (51.3)
SES		
Lower	140 (40.0)	96 (40.0)
Middle	140 (40.0)	95 (39.6)
Upper	70 (20.0)	49 (20.4)
Wheeze		
No wheeze	277 (92.6)	201 (91.8)
Wheeze	22 (7.36)	18 (8.2)
HDM SPT		
Negative	295 (96.4)	227 (95.8)
Positive	11 (3.6)	10 (4.2)
Total IgE (IU/mL) [GM^b^, (95% CI)]	83.58 (73.93, 94.50)	85.09 (74.74, 96.87)
HDM-IgE (IU/mL) [GM^b^, (95% CI)]	0.136 (.125, .147)	0.138 (.126, .151)
HDM-IgE responsiveness		
Negative^c^	203 (74.1)	170 (73.0)
Positive^c^	71 (25.9)	63 (27.0)
HDM-IgG4 responsiveness		
Negative^c^	258 (94.2)	220 (94.4)
Positive^c^	16 (5.8)	13 (5.6)
*S. mansoni* infection prevalence	319 (91.1)	225 (93.8)
*S. mansoni* infection intensity [GM epg + 1, (95% CI)]	147.42 (116.82, 186.02)	172.73 (131.61, 226.72)
Hookworm infection prevalence	271 (77.4)	193 (80.4)
Hookworm infection intensity [GM epg + 1, (95 CI)]	67.16 (50.49, 89.34)	71.75 (51.52, 99.90)
Other helminth infection	125 (35.7)	89 (37.1)^d^

Abbreviations: CI, confidence interval; epg, egg count per gram; GM, geometric mean; HDM,house dust mite allergen; IgE, immunoglobulin E; SES, socioeconomic status.

^a^ Children completing the study, ie, children who provided assent, donated at least 2 pretreatment stool samples, blood samples pretreatment and 8 weeks post-treatment, and complied with treatment procedures (n = 240).

^b^ An integer, *k*, was added to antibody readings before calculating geometric means; *k* = 1.33445 (t-IgE), *k* = 0.095 (DM-IgE).

^c^ Detection threshold calculated as the mean + 3 SD of blank wells = 0.228 (HDM-IgE), 1.22 (HDM-IgG4).

^d^ Specific prevalence of other helminth infections: *Trichuris trichiura* = 28.3%; *Hymenolepis nana* = 10.8%; *Enterobius vermicularis* = 5.4%; *Ascaris lumbricoides* = 4.6%.

### Changes in Nonspecific (α-IgE-induced) HR 8 Weeks Post-Anthelmintic Treatment

**Figure 1.**
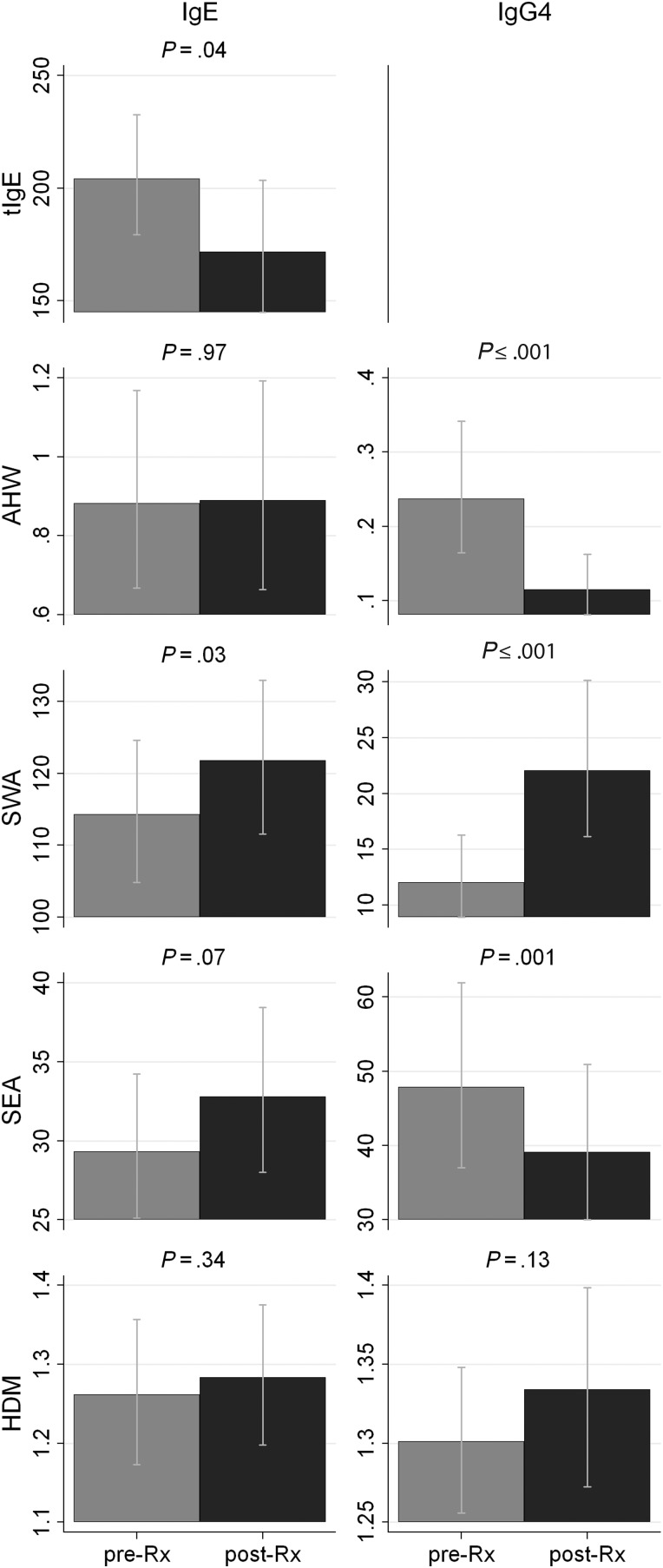
Geometric mean levels of total-IgE, and IgE and IgG_4_ to AHW, SWA, SEA, and HDM pretreatment (*gray bars*) and post-treatment (*black bars*). An integer, *k*, was added to antibody readings before calculating geometric means to remove zero values; values for *k* were as follows: total-IgE = 3.203, AHW-IgE = 0.38, AHW-IgG4 = 0.012, SWA-IgE = 79.2, SWA-IgG4 = 0.506, SEA-IgE = 9.472, SEA-IgG4 = 0.108, HDM-IgE = 0.228, HDM-IgG4 = 1.22. IgE levels are expressed in ng/mL and IgG4 levels are expressed in µg/mL. *P*-values indicate level of significance for differences between pre- and post-treatment levels. Abbreviations: AHW, adult hookworm antigen extract; HDM, house dust mite allergen; IgE, immunoglobulin E; SEA, soluble egg antigen; SWA, soluble worm antigen.

**Figure 2.**
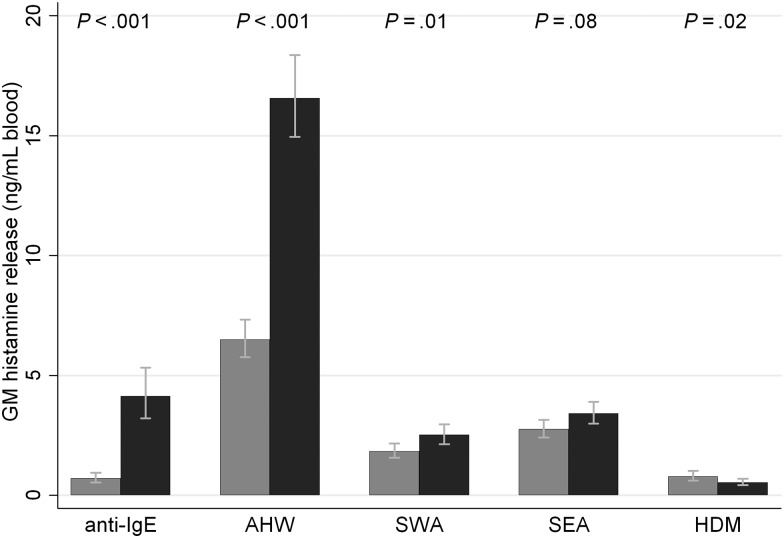
Pretreatment (*gray bars*) and post-treatment (*black bars*) in vitro basophil histamine release following culture of unwashed, whole blood with α-IgE or specific antigen. An integer, *k*, was added to histamine readings before calculating geometric means to remove zero values; values for *k* were as follows: anti-IgE = 0.064, AHW = 1.775, SWA = 0.535, SEA = 0.920, HDM = 0.096. *P*-values indicate level of significance for differences between pre- and post-treatment release. Abbreviations: AHW, adult hookworm antigen extract; HDM, house dust mite allergen; IgE, immunoglobulin E; SEA, soluble egg antigen; SWA, soluble worm antigen.

Total IgE levels decreased significantly post-treatment (Figure 1, *P* = .04). Figure 2 shows pre- and post-treatment in vitro basophil HR following incubation of whole blood with specific antigen or α-IgE; *P*-values indicate the level of significance for pre- vs post-treatment differences. Despite a decrease in tIgE, α-IgE-HR increased significantly post-treatment (Figure 2).

### Changes in Hookworm-HR 8 Weeks Post-Anthelmintic Treatment

Although AHW-IgG4 decreased significantly post-treatment (*P* < .001), AHW-IgE responses were not affected by treatment (*P* = .97, Figure 1). Despite no increase in AHW-IgE, AHW-HR increased dramatically post-treatment, from a GM of 6.50 ng/mL (95% CI, 5.77, 7.33) to a GM of 16.57 ng/mL (95% CI, 14.94, 18.36; Figure 2).

Table 2 displays pre- and post-treatment associations between plasma levels of antigen-specific IgE and HR; the significance of any change in association is also displayed. A borderline significant association between AHW-IgE and AHW-HR was observed pretreatment (*P* = .06), which strengthened and became significant post-treatment (*P* = .01); however, this change in strength of association was not significant.

**Table 2.** Pretreatment and Post-treatment Associations Between Plasma Levels of Antigen-specific IgE and Histamine Release to the Respective Antigen

Antigen	Adjusted^a^ GM Ratio (95% CI)		
	Pretreatment^b^	Post-treatment^c^	*P* Value^d^
t-IgE /anti-IgE	0.75 (.57, 1.00)*	0.91 (.75, 1.10)	.21
AHW	1.05 (1.00, 1.11)	1.06 (1.02, 1.11)**	.77
SWA	1.18 (.92, 1.50)	1.65 (1.30, 2.10)***	.04
SEA	1.03 (.92, 1.16)	1.16 (1.05, 1.29)**	.07
HDM	1.10 (.76, 1.61)^e^	1.15 (.82, 1.60)	.89

Abbreviations: CI, confidence interval; GM, geometric mean; HDM, house dust mite allergen; IgE, immunoglobulin E; SES, socioeconomic status.

^a^ Age, sex, and SES adjusted.

^b^ GM increase in pre-treatment anti-IgE-induced or antigen-induced histamine release with each unit increase in pre-treatment plasma levels of t-IgE or IgE to the same antigen. *<.05.

^c^ GM increase in post-treatment anti-IgE-induced or antigen-induced histamine release with each unit increase in post-treatment plasma levels of t-IgE or IgE to the same antigen. **<.01, ***<.001.

^d^ Significance of any change in association between non-specific or antigen-specific-HR and plasma IgE levels pre- vs post-treatment; determined by 2-level linear regression to allow for correlation within children, with time (pre- or post-treatment) as “level 1” units and child as “level 2” units, and an interaction term fitted between time and antigen-specific-IgE.

^e^ Significant interaction between HDM-IgE and hookworm infection (χ^2^(1) = 4.90, *P* = .03). Model predicted GM ratios: hookworm infection 0.22 (95% CI, .06, .79); HDM-IgE 2.65 (95% CI, 1.11, 6.33); hookworm*HDM-IgE 0.35 (95% CI, .13, .91).

### Changes in *S. mansoni*-HR 8 Weeks Post-Anthelmintic Treatment

Plasma levels of SWA-IgE and -IgG_4_ increased significantly post-treatment (*P* = .03 and < .001, respectively), SEA-IgE remained unchanged by treatment (*P* = .07), and SEA-IgG_4_ decreased significantly post-treatment (*P* = .001, Figure 1). In line with increased SWA-IgE levels, a significant post-treatment increase in SWA-HR was observed (Figure 2). SEA-HR increased marginally, but nonsignificantly, post-treatment (Figure 2).

Pretreatment, no associations were observed between SWA- or SEA-HR and plasma IgE to the respective antigen (Table 2). Post-treatment, these associations strengthened, becoming significant for both antigens. For SWA, this change in strength of association was significant (Table 2).

### Changes in House Dust Mite-HR 8 Weeks Post-Anthelmintic Treatment

Plasma HDM-IgG_4_ and -IgE levels were not significantly affected by treatment (Figure 1, *P* > .13). A small but significant decrease in HDM-HR was observed post-treatment [GM = 0.79 (95% CI, .61, 1.01) pretreatment vs GM = 0.54 (95% CI, .43, .68) post-treatment, Figure 2]. No overall association was observed between HDM-HR and HDM-IgE, either pre- or post-treatment (Table 2).

### Effect of Helminth Infection on House Dust Mite-HR

**Figure 3.**
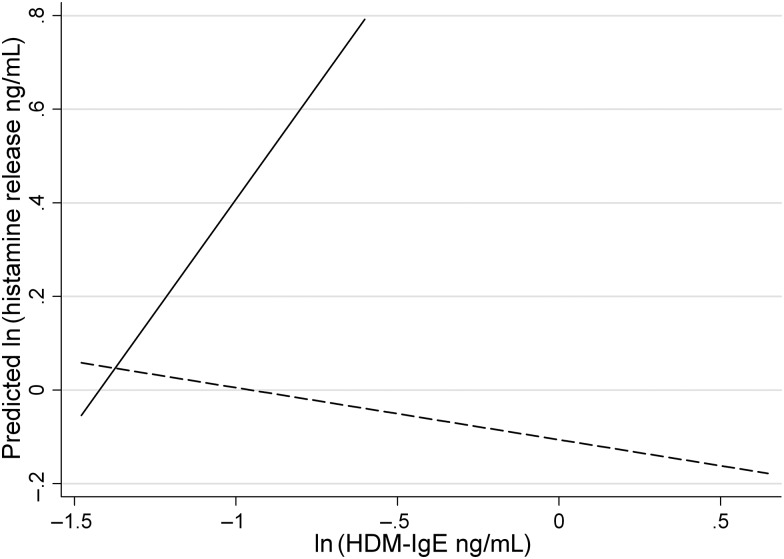
Predicted association between circulating HDM-IgE levels and house dust mite-induced histamine release among children with hookworm infection (*dashed line*) and children without hookworm infection (*solid line*). The effect modifying behaviour of pretreatment hookworm infection on the association between HDM-IgE and HDM-induced histamine release was analysed using linear regression analysis, adjusting for age, sex, and SES. A significant hookworm infection–HDM-IgE interaction was observed (χ^2^(1) = 4.90, *P* = .03). Model predicted GM ratios were as follows: hookworm infection 0.22 (95% CI, .06, .79); HDM-IgE 2.65 (95% CI, 1.11, 6.33); hookworm*HDM-IgE 0.35 (95% CI, .13, .91); age 9–10 years, 1.33 (95% CI, .66, 2.69); age 11–12 years, 0.86 (95% CI, .44, 1.68); age 13–16 years, 0.53 (95% CI, .23, 1.21); male sex, 1.50 (95% CI, .89, 2.51); ln(SES score), 0.72 (95% CI, .40, 1.29). Abbreviations: CI, confidence interval; GM, geometric mean; HDM, house dust mite allergen; IgE, immunoglobulin E; SES, socioeconomic status.

To investigate whether hookworm and/or *S. mansoni* infection modifies the immune response to environmental allergens such as HDM, we included pretreatment prevalence (hookworm) or intensity (*S. mansoni*) of infection as effect modifiers in regression analysis. Although there was no overall association between pretreatment HDM-IgE and HDM-HR (Table 2), there was evidence that hookworm infection significantly modified the influence of HDM-IgE on HDM-HR (HW infection–HDM-IgE interaction significant: LRχ^2^ = 4.78, *P* = .03). No association was observed between HDM-IgE and HDM-HR among hookworm-infected children [GM ratio_adj_ = 0.92 (95% CI, .61, 1.40)], but a strong significant association was observed among children without pretreatment infection [GM ratio_adj_ = 2.64 (95% CI, 1.10, 6.30)] (Figure 3). No significant interaction was observed between *S. mansoni* infection and HDM-IgE levels on HDM-HR (LRχ^2^ = 1.32, *P* = .25).

Changes in HDM-HR pre- and post-treatment also appeared to be influenced by pretreatment hookworm infection but also dependent on post-treatment HDM-IgE levels [pretreatment hookworm infection–post-treatment HDM-IgE interaction significant: LRχ^2^ = 4.69, *P* = .03]. Among children with pretreatment hookworm infection, HDM-HR increased post-treatment in a HDM-IgE dependent manner, with greater increases observed among children with higher post-treatment HDM-IgE levels (Table 3). There was no evidence for pretreatment *S. mansoni* infection intensity having a similar effect [pretreatment *S. mansoni* infection intensity– post-treatment HDM-IgE interaction nonsignificant: LRχ^2^ = 0.29, *P* = .59].

**Table 3.** Factors Associated With Post-treatment Changes^a^ in HDM-induced Histamine Release

	GM ratio^b^ (CI_95%_)	*P* Value
HW infected at baseline	9.19 (1.59, 53.22)	.01
Post-Rx HDM-IgE	0.34 (.10, 1.14)	.07
HW infection*Post-Rx HDM-IgE	4.19 (1.11, 15.81)	.03
SES	1.22 (.54, 2.76)	.61
9–10 y	0.64 (.24, 1.67)	
11–12 y	1.06 (.42, 2.67)	
13–16 y	1.01 (.30, 3.37)	.68
Male	0.86 (.42, 1.76)	.66

Abbreviations: CI, confidence interval; GM, geometric mean; HDM, house dust mite allergen; IgE, immunoglobulin E; SES, socioeconomic status.

^a^ Calculated as ln(post-Rx histamine release)–ln(pre-Rx histamine release).

^b^ Estimated using multiple regression analysis.

^c^ Determined by likelihood ratio tests.

### Associations Between Helminth Infection and Markers for Allergy

To investigate whether pretreatment prevalence of hookworm or intensity of *S. mansoni* infection had any influence on symptoms of allergy, we measured pretreatment SPT sensitivity to HDM and documented current wheeze. Despite 27% of children having HDM-IgE, only 4.2% of children presented with HDM-SPT sensitivity (Table 1). Although higher levels of HDM-IgE were associated with a greater risk of HDM-SPT [OR_adj_ = 1.97 (95% CI, .94, 4.12)], this relationship was nonsignificant (*P* = .09). HDM-HR was significantly associated with HDM-SPT sensitivity [OR_adj_ = 1.87 (95% CI, 1.06, 3.29), *P* = .01]. There was no evidence for either hookworm or *S. mansoni* infection modifying either of these associations (interaction term *P* > .09) or for infection influencing risk of HDM-SPT sensitivity (*P* > .13); however, IgG4 responsiveness to AHW was associated with a reduced risk of HDM-SPT sensitivity (OR_adj_ = 0.21, *P* = .03).

Similarly to SPT sensitivity, prevalence of wheeze in the last 12 months was also low, with only 8.22% of children affected. No association was observed between HDM-IgE or HDM-SPT sensitivity and risk of wheeze [OR_adj_ = 1.18 (95% CI, .58, 2.39), *P* = .65 and OR_adj_ = 1.37 (0.16, 12.04), *P* = .78, respectively], a borderline significant association was observed between HDM-HR and risk of wheeze [OR_adj_ = 1.35 (95% CI, .96, 1.91), *P* = .07]. Again, there was no evidence for either hookworm or *S. mansoni* infection influencing these relationships (interaction terms *P* > .14); however, hookworm infection and IgG4 responsiveness to AHW were both associated with a significantly decreased risk of wheeze [OR_adj_ = 0.29 (95% CI, .10, .87), *P* = .03 and OR_adj_ = 0.36 (95% CI, .13, 1.02), *P* = .05, respectively]. The protective influence of hookworm infection against wheeze appeared dose-dependent, with heavier hookworm infection intensities associated with lowest risk of wheeze [OR_adj_ = 0.80 (95% CI, .65, .98), *P* = .03]. There was no association between *S. mansoni* infection intensity and risk of wheeze [OR_adj_ = 1.05 (95% CI, .82, 1.34)].

## DISCUSSION

There is growing evidence that infection with some helminth species offers protection against allergy [13, 14]. Poor correlations between as-IgE and SPT sensitivity and as-IgE and clinical signs of allergy in helminth-infected populations [10–12] suggest that this protection may act by uncoupling asIgE from its effector mechanisms. We investigated this hypothesis in Ugandan schoolchildren resident in a *S. mansoni* and hookworm coendemic area, using whole blood assays to include both cellular and humoral factors. We observed increased nonspecific and helminth-specific HR after anthelmintic treatment, as well as stronger associations between helminth-specific IgE and helminth-specific HR. There was also evidence for hookworm infection modifying the influence of HDM-IgE on HDM-HR, and reducing risk of wheeze. Further, AHW-IgG4 responsiveness was associated with reduced risk of HDM-SPT sensitivity. Although average age and hookworm and *S. mansoni* infection prevalence were slightly higher among children completing the study, this is unlikely to have biased results; all other measured characteristics were comparable among those completing and not completing the study.

Few studies have investigated basophil function during human helminth infection. Although basophil competence has been demonstrated in human hookworm infection [32], ours is the first study to our knowledge to investigate treatment effects on basophil function in a hookworm-endemic population. Basophil function in *S. mansoni* infection has shown similar trends toward increased HR post-treatment. For example, adult *S. mansoni*-antigen-induced HR from basophils passively sensitized with human infection sera increased post-treatment and suggested that IgG4-mediated down-regulation occurred during active infection [28]. We also observed increases in α-IgE-, SEA-, and SWA-HR in washed basophils from Ugandan fishermen exposed to *S. mansoni* [27], whereas Larson et al [33] reported increases in nonspecific IgE-dependent and IgE-independent HR in washed basophils following anthelmintic treatment of a small sample of *Ascaris lumbricoides-* and *Trichuris trichiura*-infected children.

We have previously observed elevated blood basophil counts in helminth-infected individuals using precise basophil measurements (Cahen*,* unpublished data), indicating that the post-treatment increases in measured HR to anti-IgE, SWA-HR, and AHW-HR observed here, truly reflect enhanced HR rather than simply increased circulating basophil numbers. Because we used whole blood assays to measure HR, this increase in HR could reflect changes in both cellular responsiveness (ie, intracellular signal transduction) and humoral factors (ie, circulating total and specific IgE and specific IgG4).

Schistosomiasis treatment with praziquantel disrupts the tegument of adult worms, exposing otherwise sequestered allergen-like antigens such as *Schistosoma mansoni* tegumental-allergen-like 1 protein (SmTAL1). Hence, anti-worm-IgE levels often increase post-treatment [24, 34], as here. Increases in SWA-IgE could explain observed increases in SWA-HR; however it is unlikely to explain the significant increased strength of association between SWA-IgE and SWA-HR. In addition, responses to schistosome egg and adult hookworm antigens are generally not boosted by treatment [24, 34, 35]. In this study, neither SEA-IgE nor AHW-IgE was significantly affected by treatment. However, SEA-IgG4 and AHW-IgG4 levels fell post-treatment, most markedly for AHW-IgG4. This drop in IgG4 levels, together with increases in α-IgE-HR, the dramatic increases in AHW-HR, and the increased strength of association between helminth-specific IgE and helminth-specific HR post-treatment, suggests a state of immunosuppresion during chronic helminth infection, which reduces basophils' ability to respond to IgE-mediated activation. The fact that associations between IgE and IgE-mediated HR appear restored 8 weeks post-treatment, demonstrates that this helminth-mediated suppression is rapidly reversed upon anthelmintic treatment.

Unfortunately, because schistosomiasis and hookworm were treated concurrently, it is not possible to determine whether one or both parasites are responsible for this suppression. However, hookworm is clearly implicated because hookworm infection significantly modified the association between HDM-IgE and HDM-HR. No association was observed between HDM-IgE and HDM-HR among hookworm-infected children, but a strong positive association was observed among uninfected children. Further, HDM-HR boosted positively after treatment with increasing post-treatment HDM-IgE levels in children with pretreatment hookworm infection, but no post-treatment HDM-HR boosting was observed among uninfected children.

There is also evidence that this hookworm-mediated suppression of basophil responsiveness to HDM has systemic effects. Prevalence of wheeze and HDM-SPT sensitivity was low: only 8% and 4% of children affected, respectively, despite 27% having detectable HDM-IgE responses. This is comparable to other wheeze prevalence estimates in Africa [8, 36], but contrasts with regional estimates in Western Europe, North America, and Oceania of 16.7%–24.2% and 8.1%–24.6% among 13–14 and 6–7 year-olds, respectively [37]. In addition, similarly to other studies [13, 38], hookworm infection was negatively associated with current wheeze, and AHW-IgG4 negatively associated with both current wheeze and HDM-SPT sensitivity.

These negative associations with AHW-IgG4, the marked post-treatment decrease in AHW-IgG4 and simultaneous increase in HR, suggest basophil suppression may be in part IgG4-mediated. In allergen-specific immunotherapy, increases in asIgG4 are more consistently associated with clinical effectiveness than changes in IgE [39], and IgG4-containing sera from treated patients has been shown to block basophil activation [40, 41]. Because we used an unwashed system to measure HR, IgG4-mediated suppression could either be through IgG4 competing with IgE for the same epitopes, and/or through co-engagement of low-affinity IgG receptors FcγRII, found on basophils, with the high-affinity receptor FcεRI. FcγRII/FcεRI co-stimulation on human basophils has been shown to result in FcγRIIB-dependent inhibition of IgE-induced activation [22, 42]; both with FcγRII and FcεRI co-crosslinking the same antigen and with costimulation with separate antigens [43]. The increase in nonspecific (α-IgE-mediated) HR post-treatment could additionally suggest that helminth infection may also regulate basophil cell function nonspecifically, independently of specific IgG4; conversely, however, observed increases in α-IgE-HR could also be related to in vitro total-IgE mediated interference.

In conclusion, our results suggest that basophil responsiveness to IgE-mediated activation is suppressed in *S. mansoni* and hookworm coinfection but is rapidly restored by anthelmintic treatment. Hookworm-mediated suppression may additionally suppress HDM-IgE-induced HDM-HR and, in turn, allergy. We speculate that this basophil suppression may be in part IgG4-mediated. Further studies into underlying mechanisms are needed to determine if this is the case and its clinical relevance to allergy management.
